# The Fickleness of Neuronal Activation: The Role of Neuronal Stochasticity in Cognitive Impairment in the Fischer 344 Transgenic Rat Model of Alzheimer's Disease

**DOI:** 10.1002/alz70855_101742

**Published:** 2025-12-23

**Authors:** Keying Chen, Emma Pineau, Margaret M Koletar, Andrea Trevisiol, Jack Simiao He, Mary E Hill, JoAnne McLaurin, Bojana Stefanovic

**Affiliations:** ^1^ Sunnybrook Research Institute, Toronto, ON, Canada; ^2^ University of Toronto, Toronto, ON, Canada; ^3^ Sunnybrook Health Sciences Centre, Toronto, ON, Canada; ^4^ McMaster University, Hamilton, ON, Canada

## Abstract

**Background:**

Healthy brain function undergoes continuous remodeling of neuronal functional properties even in environmental and behavioral stable conditions, which likely leads to ongoing, activity‐independent synaptic changes that contribute to the stochastic responsiveness of neurons to stimulation. Reduction in volatility of neuronal responses to repeated stimulation is likely associated with the impaired information processing in brain network.

**Method:**

We inserted the ultra‐high density Neuropixels 1.0 electrode (10‐mm long, single‐shank, 384 recording channels, 70 x 24 μm cross‐section) so as to span both primary somatosensory cortex and hippocampal CA1 and CA3 regions. The electrophysiological data were sampled at 30 kHz for quantifying activity at rest and during forepaw stimulation (60s, 3Hz, 10mA). Our guiding hypothesis is that a lower trial‐dependent variation in the subset of neurons activated by a repetitive stimulus results more impaired cognition impairment in AD progression.

**Result:**

Our preliminary findings suggest that cognitively impaired rats exhibit a lower percentage of neurons responding to each stimulus, a reduced total number of responsive neurons, and decreased variability in oscillatory activity in the hippocampus compared to their cognitively maintained littermates, independent of their AD genotypes.

**Conclusion:**

The final results of this project have the potential to identify a novel target for presymptomatic AD interventions, and early stratification of AD patients by their susceptibility to dementia.

